# Serum carcinoembryonic antigen and carbohydrate antigen 19-9 as preoperative diagnostic biomarkers of extrahepatic bile duct cancer

**DOI:** 10.1093/bjsopen/zrab127

**Published:** 2021-12-22

**Authors:** Hyeong Seok Kim, Youngmin Han, Jae Seung Kang, Yoon Hyung Kang, Mirang Lee, Hee Ju Sohn, Hongbeom Kim, Wooil Kwon, Jin-Young Jang

**Affiliations:** Department of Surgery and Cancer Research Institute, Seoul National University College of Medicine, Seoul, South Korea

## Abstract

**Background:**

Serum carcinoembryonic antigen (CEA) and carbohydrate antigen (CA) 19-9 have been proposed as useful preoperative biomarkers of extrahepatic bile duct cancer (EBDC). This study investigated the accuracy of CEA and CA19-9 for preoperative diagnosis of EBDC.

**Methods:**

Patients who underwent surgery for EBDC at a tertiary centre between 1995 and 2018 were studied, and those with concurrent hepatobiliary diseases (including gallbladder cancer, intraductal papillary mucinous neoplasms of pancreas), which could affect CEA or CA19-9 levels, were excluded. The control group included patients who underwent cholecystectomy for benign gallbladder diseases during the same period. Diagnostic accuracy was determined using sensitivity, specificity and area under the receiver operating characteristic curve (AUC).

**Results:**

After excluding 23 patients, 687 patients (488 men and 199 women, mean age 65.8 years) were compared with the control group of 2310 patients. Median CEA and CA19-9 levels were 1.8 μg/l and 47.0 kU/l in patients with EBDC. CEA (cut-off 5.0 μg/l) showed AUC of 0.541, sensitivity 9.0 per cent and specificity 99.2 per cent, whereas CA19-9 (cut-off 37.0 kU/l) showed AUC of 0.753, sensitivity 56.2 per cent and specificity 94.5 per cent. Sensitivity of CA19-9 was lower in early (T stages 0–II) than advanced (T stages III and IV) cancer (47.0 *versus* 64.9 per cent), and also lower in N0 stage cancer than lymph node metastasis (50.1 *versus* 68.8 per cent).

**Conclusion:**

Serum CEA and CA19-9 showed low sensitivity limiting their usefulness as diagnostic biomarkers of EBDC.

## Teaser

To investigate the diagnostic accuracy of serum carcinoembryonic antigen (CEA) and carbohydrate antigen (CA) 19-9, the authors analysed 710 patients for extrahepatic bile duct cancer. CEA showed relatively low, but CA 19-9 showed moderate sensitivity for the diagnosis; sensitivity was lower in early T stage and N0 cancer.

## Introduction

Extrahepatic bile duct cancer (EBDC) includes hilar and distal bile duct cancers^1^. Although surgical resection is associated with long-term survival, this malignancy has a relatively poor prognosis; the overall 5-year survival rates range from 32.2 to 48.3 per cent^1–3^. Patients with low T stage cancers or those without lymph node metastasis have better prognoses^4^^,^^5^. Early detection of this cancer is challenging because incidence is low, making imaging tests for population screening not cost-effective.

Tumour markers may serve as useful non-invasive biomarkers for screening purposes. Carcinoembryonic antigen (CEA) and carbohydrate antigen (CA) 19-9 have been widely used as known tumour markers used in patients with pancreatobiliary neoplasms. A recent study found that preoperative serum CEA and CA19-9 levels can predict the resectability of cholangiocarcinoma^6^, while another found that elevated serum CEA and CA19-9 levels were associated with moderate sensitivity, true negativity rates and accuracy for the diagnosis of cholangiocarcinoma^7^. The authors suggested that serum CA19-9 was an effective tumour marker in determining the resectability and monitoring treatment effects.

Few studies have investigated the diagnostic accuracy of these tumour markers in patients with EBDC. As CEA and CA19-9 may be elevated in patients with several benign pancreatobiliary diseases and other gastrointestinal malignancies^8–11^, this study investigated the usefulness of CEA and CA19-9 as preoperative diagnostic biomarkers of EBDC.

## Methods

This study included patients who underwent surgery for EBDC at Seoul National University Hospital, and excluded patients with gallbladder cancer. The control group included patients who underwent cholecystectomy for benign gallbladder diseases during the same period.

Peripheral venous blood samples were obtained preoperatively, and serum CEA and CA19-9 levels were measured using an electrochemiluminescence immunoassay. Standardized cut-off levels identified for CEA and CA19-9 were 0.1–5.0 μg/l and 1.0–37.0 kU/l respectively. Subgroup analysis was performed to determine the effects of obstructive jaundice or cholangitis, which were defined as serum total bilirubin levels of at least 51.3 μmol/l. This value was previously used as a cut-off value of jaundice in other studies^12^^,^^13^. Preoperative biliary drainage procedures, including endoscopic retrograde biliary drainage, endoscopic nasobiliary drainage, percutaneous transhepatic biliary drainage or percutaneous transhepatic gallbladder drainage, were performed to alleviate symptomatic jaundice.

Among patients with EBDC who were initially enrolled, patients with concurrent pancreatobiliary diseases (which could affect the serum CEA or CA19-9 levels), were excluded.

All data were obtained from and analysed at the Department of Surgery, Seoul National University, Korea. This study was approved by the Institutional Review Board of Seoul National University Hospital (approval number: 1812–002-989).

All statistical analyses were performed using the R software, version 3.6.3 (The R Foundation for Statistical Computing, Vienna, Austria). Continuous variables are reported as medians (i.q.r.). Log transformation was used in cases of skewed distribution of serum CEA and CA19–9 levels. The area under the receiver operating characteristic curve (AUC) was used to assess the predictive power of the tumour markers based on their cut-off levels. Sensitivity, specificity, accuracy, and positive and negative predictive values of each threshold value were measured to evaluate predictive ability. Accuracy was defined as the percentage of correctly classified instances ((true positive + true negative)/(true positive + true negative + false positive + false negative)).

## Results

### Demographics and clinicopathological findings

After excluding 23 patients with concurrent pancreatobiliary diseases from 710 patients who underwent surgery between 1995 and 2018, 687 patients were included in the final analysis. The demographic and clinicopathological characteristics of these patients are shown in *Table 1*. The mean patient age was 65.8 years, and the male : female ratio was 2.45 : 1. Median serum CEA and CA19-9 levels were 1.8 (i.q.r. 1.2–2.9) μg/l and 47.0 (i.q.r. 17.2–140.9) kU/l, respectively, and the percentages of patients with elevated serum CEA (greater than 5.0 μg/l) and CA19-9 (greater than 37.0 kU/l) were 9.0 and 56.2 per cent, respectively. The median serum total bilirubin level at the time of tumour marker measurement was 1.6 (i.q.r. 0.9–3.5) mg/dl, and the percentage of patients with elevated bilirubin (greater than 5.0 mg/dl) was 15.5 per cent.

**Table 1:** Demographic and clinicopathological characteristics of patients with extrahepatic bile duct cancer

Characteristic	Patients (*n* = 687)
Age*	65.8 (9.0)
**Sex (M : F)**	488 : 199
BMI*	23.2 (2.9)
**CEA (μg/l**)†	1.8 (1.2–2.9)
**CEA >5 μg/l**	62 (9.0)
**CA19-9 (kU/l)**†	47.0 (17.2–140.9)
**CA19-9 >37 kU/l**	386 (56.2)
**Total bilirubin (µmol/l**)†	1.6 (0.9–3.5)
**Total bilirubin >51.3 µmol/l**	197 (28.7)
**Preoperative biliary drainage**	
ERBD	345 (50.2)
PTBD	239 (34.8)
ENBD	107 (15.6)
PTGBD	13 (1.9)
**Operation**	
Whipple, PPPD	618 (90.0)
Hilar resection	15 (2.2)
HPD	4 (0.6)
Total	3 (0.4)
Bypass	28 (4.1)
Others	19 (2.8)
**T stage**	
0, 1	92 (13.4)
2	206 (30.0)
3	339 (49.3)
4	11 (1.6)
**N stage**	
0	427 (62.2)
1	199 (29.0)
2	19 (2.8)

Values in parentheses are percentages unless indicated otherwise; *values are indicated mean (SD), †values are indicated median (IQR). CA19–9, carbohydrate antigen 19–9; CEA, carcinoembryonic antigen; HPD, hepatopancreatoduodenectomy; ENBD, endoscopic nasobiliary drainage; ERBD, endoscopic retrograde biliary drainage; PPPD, pylorus-preserving pancreatoduodenectomy; PTBD, percutaneous transhepatic biliary drainage; PTGBD, percutaneous transhepatic gallbladder drainage.

Among all the patients investigated, 636 patients (92.6 per cent) required preoperative biliary drainage; the most common method used was endoscopic retrograde biliary drainage in 345 patients (50.2 per cent), followed by percutaneous transhepatic biliary drainage in 239 patients (34.8 per cent) and endoscopic nasobiliary drainage in 107 patients (15.6 per cent). Operations with curative intent were performed in 642 patients (93.4 per cent). In total, 545 patients (79.3 per cent) were diagnosed with T stage II or III; 427 patients (62.2 per cent) showed no lymph node metastasis.

In the control group of 2310 patients, the mean patient age was 54.3 years, and the male : female ratio was 0.89 : 1. The median serum CEA and CA19-9 levels were 1.4 (i.q.r. 1.0–2.0) μg/l and 7.5 (i.q.r. 2.8–15.6) kU/l respectively, and the percentages of patients with elevated serum CEA (greater than 5.0 μg/l) and CA19-9 (greater than 37.0 kU/l) were 0.8 and 5.5 per cent respectively.

### Distribution of tumour markers in patients with extrahepatic bile duct cancer and the control group

The serum CEA (0.6 *versus* 0.4 μg/l, *P*<0.001) and CA19-9 (47.0 *versus* 7.5 kU/l, *P*<0.001) levels were significantly higher in the cancer than in the control group. Additionally, the percentage of patients with elevated serum CEA (9.0 *versus* 0.8 per cent, *P*<0.001) and CA19-9 (56.2 *versus* 5.5 per cent, *P*<0.001) levels was higher in the cancer group than in the control group.

**Fig. 1:**
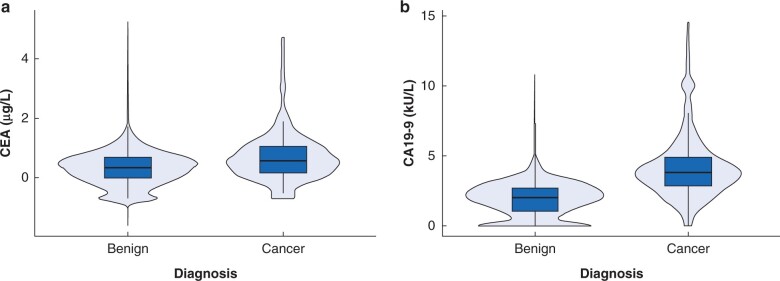
Distribution of prospective biomarkers in patients with extrahepatic bile duct cancer and the control group **a** Carcinoembryonic antigen (CEA). Median CEA 0.4 *versus* 0.6 (1.4 *versus* 1.8 μg/l) (*P* < 0.001) Mann-Whitney U test. **b** Carbohydrate antigen 19-9 (CA19-9). Median CA19-9 2.0 *versus* 3.9 (7.5 *versus* 47.0 kU/l) (*P* < 0.001) Mann-Whitney U test.

*Fig. 1* shows the distribution of tumour markers in the cancer and control groups with log-transformed serum CEA and CA19-9 levels. Although a statistically significant intergroup difference was observed in serum CEA levels, the distributions were similar between groups. CA19-9 was more distinguishable than CEA; however, a large percentage of patients overlapped, and the cut-off level for CA19-9 could not be determined definitively between groups.

### Diagnostic accuracy of CEA and CA19-9 based on cut-off levels for the diagnosis of extrahepatic bile duct cancer

**Fig. 2:**
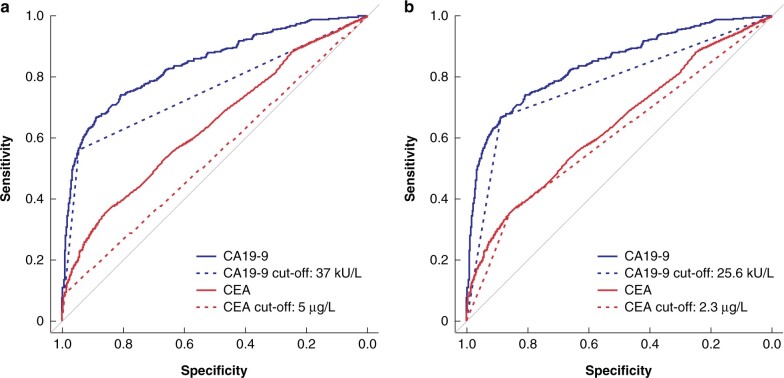
Diagnostic accuracy using the receiver operating characteristic curve and area under the curve values of prospective biomarkers based on cut-off levels **a** Carcinoembryonic antigen (CEA) cut-off level 5.0 μg/l and carbohydrate antigen 19-9 (CA19-9) cut-off level 37.0 kU/L. **b** Optimal cut-off levels of CEA and CA19-9.

*Table 2* and *Fig. 2* show the diagnostic accuracy of these markers represented by the AUC, sensitivity, specificity, accuracy, as well as the positive and negative predictive values, based on cut-off levels. The AUC of CEA for cancer prediction was 0.641. The AUC, sensitivity and specificity were 0.541, 9.0 per cent and 99.2 per cent, respectively, at a cut-off level of 5.0 μg/l. The optimal cut-off level that maximized the AUC was 2.3 μg/l.

**Table 2:** Accuracy of serum carcinoembryonic antigen and carbohydrate antigen 19–9 as diagnostic biomarkers of cancer based on cut-off levels

Biomarker	**Cut-off (μg/l**)	AUC	Sensitivity (%)	Specificity (%)	Accuracy (%)	PPV (%)	**NPV (%)**
**CEA**	5	0.541	9.0	99.2	78.5	77.5	78.6
	2.3	0.606	35.5	85.7	74.2	42.4	81.7
**CA19–9**	37	0.753	56.2	94.5	85.7	75.2	87.9
	25.6	0.777	66.5	88.9	83.8	64.1	89.9

AUC, area under the curve; CA19–9, carbohydrate antigen 19-9; CEA, carcinoembryonic antigen; NPV, negative predictive value; PPV, positive predictive value.

The AUC of CA19-9 for cancer prediction was 0.841. The AUC, sensitivity and specificity were 0.753, 56.2 per cent and 94.5 per cent respectively, at a cut-off level of 37.0 kU/l (*Table 2*). The optimal cut-off level for CA19-9 was 25.6 kU/l.

### Effect of obstructive jaundice or cholangitis on CEA and CA19-9 for the diagnosis of extrahepatic bile duct cancers

Subgroup analysis was based on serum total bilirubin levels (51.3 µmol/l) to determine the effect of obstructive jaundice. A total of 197 patients (28.7 per cent) showed elevated bilirubin levels, and the AUCs of serum CEA and CA19-9 in these patients were 0.565 and 0.820 respectively (*Table 3*). When compared with the 447 (65.1 per cent) patients with levels of bilirubin less than 51.3 µmol/l, those with elevated bilirubin showed higher sensitivity of CEA (13.7 *versus* 6.5 per cent) and CA19-9 (69.5 *versus* 48.8 per cent).

**Table 3:** Subgroup analysis of serum carcinoembryonic antigen and carbohydrate antigen 19-9 based on the serum total bilirubin levels and the T and N stages of The American Joint Committee on Cancer staging system

		AUC	Sensitivity (%)	Specificity (%)	PPV (%)	NPV (%)
**Total bilirubin**						
≥51.3 µmol/l (*n* = 197)	CEA	0.565	13.7	99.2	60.0	93.1
	CA19-9	0.820	69.5	94.5	51.9	97.3
<51.3 µmol/l (*n* = 447)	CEA	0.529	6.5	99.2	61.7	84.6
	CA19-9	0.716	48.8	94.5	63.2	90.5
**T stage**						
0, I, II (*n* = 298)	CEA	0.538	8.4	99.2	58.1	89.4
	CA19-9	0.707	47.0	94.5	52.4	93.3
III, IV (*n* = 350)	CEA	0.540	8.9	99.2	63.3	87.8
	CA19-9	0.797	64.9	94.5	64.1	94.7
**N stage**						
0 (*n* = 427)	CEA	0.541	8.9	99.2	67.9	85.5
	CA19-9	0.723	50.1	94.5	62.8	91.1
I, II (*n* = 218)	CEA	0.540	8.7	99.2	51.4	92.0
	CA19-9	0.817	68.8	94.5	54.2	97.0

AUC, area under the curve; CA19-9, carbohydrate antigen 19-9; CEA, carcinoembryonic antigen; NPV, negative predictive value; PPV, positive predictive value.

### Association between tumour markers and the American Joint Committee on Cancer staging system for the diagnosis of extrahepatic bile duct cancer

**Fig. 3:**
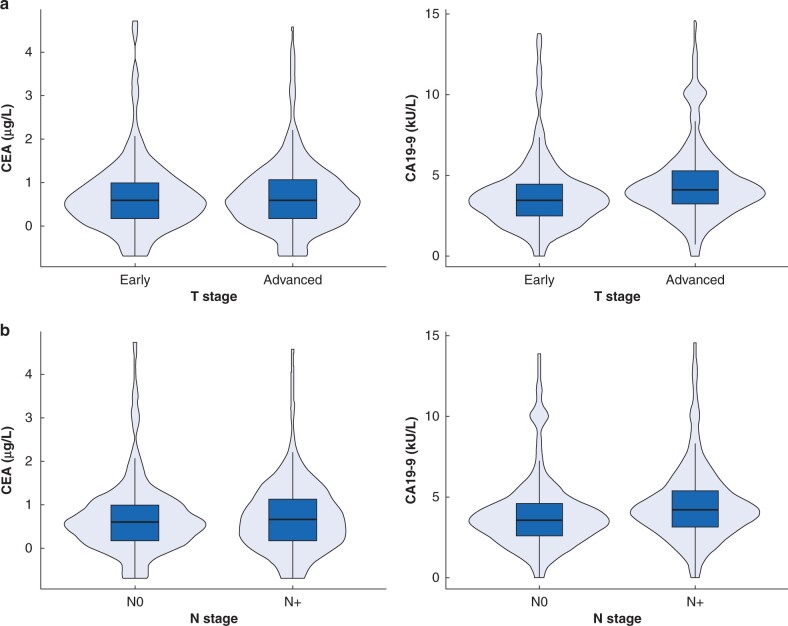
Distribution of prospective biomarkers Distribution based on the American Joint Committee on Cancer staging system **a** T (median carcinoembryonic antigen (CEA) 0.6 *versus* 0.6 (1.8 *versus* 1.8 μg/l) (*P* < 0.798); median carbohydrate antigen 19-9 (CA19-9) 3.5 *versus* 4.1 (31.6 *versus* 61.6 kU/l) (*P* < 0.001)), and **b** N stage (median CEA 0.6 *versus* 0.6 (1.8 *versus* 1.9 μg/l) (*P* < 0.592); median CA19-9 3.6 *versus* 4.2 (37.1 *versus* 67.9 kU/l) (*P* < 0.001)), in patients with early- and advanced-stage cancer.

Subgroup analyses based on the American Joint Committee on Cancer (AJCC) T and N stages to confirm the diagnostic accuracy of the markers indicated that CEA showed relatively low diagnostic accuracy (AUC 0.540) and sensitivity of 8.9 per cent, even in patients with locally advanced (T stages III and IV) disease (*Table 3*, *Fig. 3*). Comparison between patients with N0 cancer and those with lymph node metastasis revealed that CEA also showed low AUCs of 0.540 and sensitivity of 8.7 per cent in patients with lymph node metastasis.

CA19-9 had better predictive ability, but lower AUCs (0.707 *versus* 0.797) and sensitivity (47.0 *versus* 64.9 per cent) in patients with early T stages (0–II) than in those with advanced T stages (stages III and IV). Patients without lymph node metastasis (N0) showed a lower AUC (0.723 *versus* 0.817) and sensitivity (50.1 *versus* 68.8 per cent) than those with lymph node metastasis.

### Subgroup analyses of diagnostic accuracy based on cut-off levels and the American Joint Committee on Cancer staging system in patients with serum total bilirubin below 51.3 µmol/l

Subgroup analyses performed for 447 patients with serum total bilirubin below 51.3 µmol/l indicated that at a cut-off level of 5.0 μg/l, the AUC and sensitivity were 0.529 and 6.5 per cent respectively (*Table S1*). The optimal cut-off level that maximized the AUC was 2.3 μg/l. The AUC and sensitivity were 0.716 and 48.8 per cent respectively at a cut-off level of 37.0 kU/l. The optimal cut-off level for CA19-9 was 18.8 kU/l.

Subgroup analyses based on the AJCC T and N stages for these patients (*Table S2*) showed that CEA still exhibited low AUCs (0.533 and 0.533 respectively) and sensitivity (7.3 and 7.3 per cent respectively), both in patients with advanced T stage and lymph node metastasis. CA19-9 still showed better predictive ability in patients with serum total bilirubin below 51.3 µmol/l. However, in patients with early T stage (0–II) disease and those without lymph node metastasis (N0), CA19-9 showed relatively lower AUCs (0.669 and 0.680 respectively) and sensitivity (39.3 and 41.5 per cent respectively).

## Discussion

This study has shown that CEA had a low AUC (0.541) and poor sensitivity (9.0 per cent) for the preoperative diagnosis of EBDC. In contrast, CA19-9 showed a moderate AUC (0.753) and sensitivity (56.2 per cent). Subgroup analyses based on the AJCC staging system revealed that for CEA, AUC and sensitivity remained low even in advanced T and N stage cancers. With regard to CA19-9, patients with early T stage and N stage cancer showed lower AUC and sensitivity. The results remained similar even in subgroup analysis when non-jaundiced (serum total bilirubin levels less than 51.3 µmol/l) patients were analysed separately.

CEA is a well known prognostic biomarker in patients with colorectal cancers^14^^,^^15^, but it appears to have a limited role in patients with bile duct cancers. The present study showed that only 9 per cent of patients with cancer had elevated serum CEA levels, and low AUC and sensitivity, with the distribution of serum CEA being similar in patients with cancer and benign disease (*Fig. 1*). CEA alone may not distinguish accurately between cancer and benign disease. CEA does not seem to be a useful diagnostic biomarker of EBDC owing to low sensitivity.

Previous studies have also examined the value of serum CA19-9 levels^7^^,^^16^. A recent study reported 64 per cent sensitivity and 69 per cent specificity of CA19-9 for the diagnosis of pancreatic cancer and cholangiocarcinoma^16^, although only 15 patients (3 per cent) had cholangiocarcinoma. Another study showed significantly higher levels of bilirubin in patients with cancer than in patients with benign biliary disease (47.4 ± 2.1 *versus* 8.3 ± 0.7 µmol/l, *P* < 0.05), although there was no correlation found between CA19-9 and bilirubin^7^. In the present study, 687 cancer patients were included and subgroup analyses for patients with total bilirubin below 51.3 µmol/l were performed to reduce the effect of bilirubin on CA19-9. CA19-9 showed only moderate sensitivity for diagnosis of cancer, again there was an overlap in the distribution of CA19-9 with the control group with benign disease (*Fig. 1*), despite higher serum CA19-9 levels in the cancer group. As a result, it was difficult to determine a definitive cut-off level to distinguish between cancer and benign disease. Moreover, patients with early T or N stage cancer showed lower sensitivity. Owing to its low diagnostic accuracy in early-stage cancer, CA19-9 is not useful for screening of EBDC.

Novel diagnostic biomarkers of bile duct cancer, including the Mac-2-binding protein and receptor-binding cancer antigen expressed on SiSo cells have been reported previously^17^^,^^18^, but seem to have remained uninvestigated.

The present study has a number of limitations. CA19-9 is a sialylated Lewis A blood group antigen, and it is known to be expressed by 95 per cent of the population, causing false-negative results in some patients^19^. Future studies with the measurement of Lewis A antigen will be required. This study was performed at a tertiary hospital where many patients had already received biliary drainage before attending. Changes in tumour markers according to the bilirubin levels could not be confirmed. The control group was younger with a greater proportion of females than the patient group.

Serum CEA and CA19-9 levels showed limited usefulness as diagnostic biomarkers for EBDC and CA19-9 also showed low sensitivity for the detection of early-stage cancer and making it of no value for screening. Biomarkers with high diagnostic accuracy in patients with EBDC are still needed.

## Supplementary Material

zrab127_Supplementary_Data
