# Supplementary material for: Serum carcinoembryonic antigen and carbohydrate antigen 19-9 as preoperative diagnostic biomarkers of extrahepatic bile duct cancer
Source: BJS Open. 2021 Dec 22;5(6):zrab127. doi: 10.1093/bjsopen/zrab127 (PMC8693162; doi:10.1093/bjsopen/zrab127)
Supplement: zrab127_Supplementary_Data [file zrab127_supplementary_data.docx]

**Table S1** Subgroup analysis of diagnostic accuracy of serum carcinoembryonic antigen and carbohydrate antigen 19-9 based on cut-off levels in patients with serum total bilirubin < 51.3 µmol/L

|  | Cut-off | AUC | Sensitivity | Specificity | Accuracy | PPV | NPV |
| --- | --- | --- | --- | --- | --- | --- | --- |
| CEA | 5 | 0.529 | 6.5 | 99.2 | 84.2 | 61.7 | 84.6 |
|  | 2.3 | 0.580 | 30.4 | 85.7 | 76.7 | 29.1 | 86.4 |
| CA19-9 | 37 | 0.716 | 48.8 | 94.5 | 87.1 | 63.2 | 90.5 |
|  | 18.8 | 0.742 | 67.6 | 80.8 | 78.6 | 40.5 | 92.8 |

Abbreviations: AUC, area under the curve; CA19-9, carbohydrate antigen 19-9; CEA, carcinoembryonic antigen; NPV, negative predictive value; PPV, positive predictive value

**Table S2** Subgroup analysis of diagnostic accuracy of serum carcinoembryonic antigen and carbohydrate antigen 19-9 based on the T and N stages of the American Joint Committee on Cancer staging system in patients with serum total bilirubin < 51.3 µmol/L

|  |  | AUC | Sensitivity | Specificity | PPV | NPV |
| --- | --- | --- | --- | --- | --- | --- |
| T stage |  |  |  |  |  |  |
| 0, 1, 2 (n = 206) | CEA | 0.523 | 5.3 | 99.2 | 37.9 | 92.2 |
|  | CA19-9 | 0.669 | 39.3 | 94.5 | 38.9 | 94.6 |
| 3, 4 (n = 218) | CEA | 0.533 | 7.3 | 99.2 | 47.1 | 91.9 |
|  | CA19-9 | 0.766 | 58.7 | 94.5 | 50.2 | 96.0 |
| N stage |  |  |  |  |  |  |
| 0 (n = 272) | CEA | 0.527 | 6.3 | 99.2 | 48.6 | 90.0 |
|  | CA19-9 | 0.680 | 41.5 | 94.5 | 47.1 | 93.2 |
| 1, 2 (n = 150) | CEA | 0.533 | 7.3 | 99.2 | 37.9 | 94.3 |
|  | CA19-9 | 0.786 | 62.7 | 94.5 | 42.5 | 97.5 |

Abbreviations: AUC, area under the curve; CA19-9, carbohydrate antigen 19-9; CEA, carcinoembryonic antigen; NPV, negative predictive value; PPV, positive predictive value
